# Evaluation of Safety and Probiotic Properties of *Weissella* spp. in Fermented Vegetables From Xi'an, Shaanxi, China

**DOI:** 10.1002/fsn3.4592

**Published:** 2024-12-02

**Authors:** Chen Liu, Chao An, Jingjing Zhang, Yao Liu, Qiwen Zhang, Hao Ding, Saijian Ma, Wenjiao Xue

**Affiliations:** ^1^ Shaanxi Institute of Microbiology Xi'an China; ^2^ Shaanxi Key Laboratory of Qinling Ecological Security Shaanxi Academy of Sciences Xi'an China

**Keywords:** fermented vegetable, probiotic properties, safety, Shaanxi, *Weissella* strains

## Abstract

The genus *Weissella*, commonly found in fermented foods, is a significant group of lactic acid bacteria (LAB) with potential probiotic properties. Several *Weissella* strains have been proposed as probiotics due to their biotechnological capabilities. However, a few strains may exhibit opportunistic pathogenic behavior, which restricts the widespread use of all *Weissella* strains in food applications. This study sought to expand our understanding of the biotechnological capabilities of *Weissella* spp. by examining the safety and functional characteristics of strains isolated from spontaneous fermentation. In this investigation, nine *Weissella* strains were evaluated for their safety and probiotic potential. The safety assessment revealed that the antibiotic resistance profiles of strains 16‐2, 38‐3, 69‐3, 91‐3, 91‐5, 104‐4, and 106‐5 were comparable or superior to the reference strain LGG. Hemolytic activity and ammonia production were also evaluated, but no positive results were observed. Further probiotic experiments demonstrated that strain 91‐5 exhibited superior performance in several areas, including survival rates in simulated gastrointestinal fluids, cell surface properties (hydrophobicity and adhesion to Caco‐2 cells), ABTS+ scavenging ability, antimicrobial activity, and cholesterol assimilation in vitro. Additionally, strain 104‐4 produced an exopolysaccharide (EPS) yield of 35.11 g/L after 48 h of culture in MRS‐sucrose (60 g/L) medium, surpassing most previously reported values. These findings suggest that strains 91‐5 and 104‐4 show promise as potential probiotic candidates for the development of new functional food supplements. Furthermore, this research expands the theoretical basis for considering *Weissella* strains as novel probiotics.

## Graphical abstract

The nine strains of *Weissella* are compliant with international safety standards and have the potential to attain Generally Recognized as Safe (GRAS) status. These *Weissella* strains exhibits diverse and significant probiotic potential, could be a source of novel probiotics. Strain 91‐5 exhibited the most promising potential as a probiotic microorganism, while strain 104‐4 demonstrated the highest yield of EPS (35.11 ± 0.59 g/L), could be further used as a probiotic candidate for new functional food supplements.

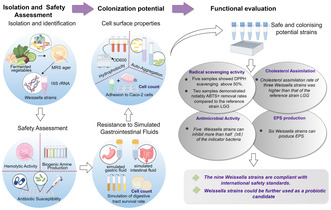

## 1 Introduction

Fermented vegetables possess a longstanding history in China, with their origins tracing back several millennia. In Shaanxi Province, located in northwestern China, fermentation has been traditionally employed to preserve vegetables throughout the winter months. This enduring practice has culminated in the creation of diverse fermented vegetable varieties, which serve as important sources of probiotics (Liu et al. 2019; Zhang et al. 2018). Lactic acid bacteria (LAB), the most important microorganisms isolated from fermented vegetables (Ma et al. 2019; Rusu et al. 2019), are recognized as Generally Recognized as Safe (GRAS) in the USA and have a Qualified Presumption of Safety (QPS) in the EU due to their long‐standing use in food (Nguyen et al. 2015). A wide range of LAB from fermented foods has been extensively studied for their probiotic characteristics, including antioxidant properties (Chuang et al. 2016), cholesterol‐lowering effects (Zhang et al. 2020), regulation of intestinal flora balance (Wiese et al. 2015), and immune enhancement (Yeganegi et al. 2011), etc. all of which contribute to multiple health benefits. Nowadays, there are more than 20 LAB species being utilized as commercial starter cultures and nutraceuticals. Among these, *Lactococcus lactis, Lactobacillus plantarum*, *Lactobacillus sakei* , *Lactobacillus delbrueckii* , *Streptococcus thermophilus*, *Leuconostoc mesenteroides* , and *Pediococcus pentosaceus* are the most widely used (Remize and Fessard 2017).

Similar to other commercial LAB species (Stenman et al. 2015), *Weissella* strains have been identified as dominant strains in many types of fermented foods (Jung and Lee 2020; Mun and Chang 2020), primarily appearing during the prophase and metaphase of fermentation (Sun et al. 2021). The *Weissella* genus, belonging to the *Leuconostocaceae* family, was established by Collins et al. in 1993 based on phylogenetic tree correlations and a unique murein type. The genus comprises about 21 species of gram‐positive, non‐spore‐forming, heterofermentative, cocci, or rod‐shaped bacteria (Mun and Chang 2020). Although some *Weissella* strains may act as opportunistic pathogens (Fairfax, Lephart, and Salimnia 2014), they have become a focal point of probiotic research due to their significant probiotic properties and the growing market demand for novel probiotics. Species such as *W. cibaria, W* *. confusa* , and *W. paramesenteroides* have been extensively studied for their probiotic potential (Pabari et al. 2020; Teixeira et al. 2021). For instance, *Weissella* strains produce antimicrobial metabolites that protect against bacterial and fungal infections (Li et al. 2017; Wang et al. 2017). *W. cibaria* strain CMU exhibits strong antimicrobial activity against oral pathogens (Jang et al. 2016) and *W. cibaria* PL9023 inhibits the growth of vaginal pathogens (Lee 2005); Multiple studies have documented the production of EPS by *Weissella* spp. at levels ranging from 10 mg/L to 1 g/L, with a high molecular mass of 10^6^ Da. (Ahmed et al. 2012; Tingirikari, Kothari, and Goyal 2014). Additionally, research has indicated that *W. koreensis* OK1‐6 exhibits potential anti‐obesity and cholesterol‐lowering effects by reducing epididymal fat pad weight, triglyceride, and cholesterol levels in obese mice. (Remize and Fessard 2017).

Previous research has indicated that probiotic properties and health benefits are generally “strain‐specific” and may not be extrapolated to other strains within the same genus or species (Pino, Russo, et al. 2019). The objective of this research was to assess the safety and efficacy of *Weissella* strains obtained from naturally fermented vegetables in accordance with international regulations and to gather credible information to support the potential designation of Generally Recognized as Safe (GRAS) status for the species. Specifically, the study initially investigated the antibiotic susceptibility, ammonia production capability, and hemolytic activity of the isolates, followed by assessments of their survival in simulated digestive fluids, pH value, growth performance, hydrophobicity, auto‐aggregation, adhesion to Caco‐2 cells, cholesterol assimilation, EPS production, antioxidant capacity, and antimicrobial capacity in vitro.

## 2 Materials and Methods

### 2.1 Isolation and Identification

Nine *Weissella* strains (Table 1) isolated from fermented vegetables purchased from various local markets in Shaanxi were cultured in MRS broth for 16–18 h at 37°C. Genus‐level identification was accomplished through Sanger sequencing of the 16S rRNA gene (Liu et al. 2022).

**TABLE 1:** Identification of *Weissella* strains isolated from spontaneous fermented vegetables in Shaanxi, China.

Strain	Origin	Identification (references)	GenBank accession no.	Percentage similarity (%)
69‐3	HuaRun pickled Chinese cabbage	*W. confusa* strain 3881	OP493236	100.00
91‐3	HuaRun pickled cabbage	*W. confusa* strain 6249	OP493237	99.80
91‐5	HuaRun pickled cabbage	*W. confusa* strain 2992	OP493238	99.86
92‐2	JiuRunju pickled chili	*W. confusa* strain SX6R5	OP493239	99.73
104‐4	HongXing pickled cabbage	*W. confusa* strain XT7‐7	OP493240	99.93
106‐5	QiXiangge pickled cowpea	*W. confusa* strain 3881	OP493241	99.86
16‐2	Mei Long pickled cabbage	*W. paramesenteroides* strain 3151	OP493242	99.52
38‐3	Yuan Gen xiang pickled leaf mustard	*W. viridescens* strain 6996	OP493243	99.86
131‐3	Yong Jian pickled ginger	*W. viridescens* strain 6996	OP493244	98.85

### 2.2 Acid Production and Growth Properties

After two passages, strain fermentation solution was inoculated at 1% (v/v) in MRS broth and standing culture for 24 h at 37°C, with pH measurements taken every two hours using a pH meter (Sartorius Basic Meter PB‐10, Germany). Bacterial biomass was quantified at 600 nm using a microplate reader (Synergy H1, BioTek) every two hours automatically. Each experiment was conducted at least three times on separate days to ensure reproducibility. (Remize and Fessard 2017).

### 2.3 Probiotic Properties

#### 2.3.1 Survival Rate in Simulated Gastrointestinal Fluids

The survival rate of the *Weissella* strain in the gastrointestinal tract aligns with the methodology employed in our prior study. (Liu et al. 2022), with minor modifications. Briefly, after two passages, cell numbers were subsequently standardized to 10^8^ colony‐forming units per milliliter (CFU/mL) using simulated gastric fluid (Ga) or simulated intestinal fluid (In). The resulting mixture was then incubated in a 37°C for 3 h for Ga or 4 h for In, to simulate the gastrointestinal transit time in humans. The survival rate was determined using the plate counting method.

#### 2.3.2 Cell Surface Characteristics

##### 2.3.2.1 Hydrophobicity

Cell surface hydrophobicity was assessed using chloroform. Thalli were isolated and collected via centrifugation, followed by resuspension of the cell pellets in PBS (pH 7.20) with an adjusted OD 600 of 0.4 ± 0.002 (designated as *A*_0_). Subsequently, one milliliter of chloroform was introduced to 3 mL of the cell suspension, which was then vortexed for 30 s. After allowing the mixture to stand at room temperature for 30 min, the aqueous phase (1 mL) was meticulously transferred to a cuvette, and the absorbance (designated as *A*_*X*_) was recorded at 600 nm. The hydrophobicity rate was determined using the following equation:

$$\text{Hydrophobicity}\;\left(\%\right)=\left(\frac{A_{0}-A_{X}}{A_{0}}\right)\times 100\%$$

##### 2.3.2.2 Auto‐Aggregation

The auto‐aggregation capabilities of nine *Weissella* strains were assessed following the methodology outlined by Choi et al. (2018). *Weissella* thalli were harvested through centrifugation, 8228 g, 15 min at 4°C, and the cell concentration was adjusted to 10^8^ CFU/mL using PBS buffer (pH 7.2). A 4 mL cell suspension was statically incubated at 37°C for 2–28 h. Subsequently, the supernatant was mixed with three times the volume of PBS. The OD600 values of the resultant mixture were assessed in order to quantify the degree of auto‐aggregation.

$$\text{Aggregation}\;\left(\%\right)=\left(\frac{A_{0}-A_{t}}{A_{0}}\right)\times 100\%$$

*A*_0_ represents t OD600 value at 0 h; *A*_*t*_ represents the OD600 value at different sampling times.

##### 2.3.2.3 Adhesion to Caco‐2 Cells

The adhesion of bacteria to Caco‐2 cells was evaluated following the methodology outlined by Liu et al. (2022). In brief, Caco‐2 cells were cultured in 24‐well cell culture plates at 37°C in a 5% CO_2_ environment for 15 days to achieve cell differentiation. Subsequently, one milliliter of *Weissella* DMEM resuspension (10^8^ CFU/mL) was added to each well and co‐incubated with the Caco‐2 monolayers for 1 h at 37°C in a 5% CO_2_ atmosphere. Non‐attached *Weissella* cells were removed by washing twice with pre‐warmed DPBS (pH 7.2, Procell, China). Adherent bacteria were then detached using DPBS containing 1% (v/v) Triton X‐100. Adhesion capacity was determined using the plate counting method.

#### 2.3.3 Antimicrobial Activity

The antibacterial activity of *Weissella* strains was assessed using the plate diffusion method. Seven indicator bacteria ( *Salmonella paratyphi* B CMCC50094, *Shigella flexneri* CMCC51574, *Staphylococcus aureus* subsp. aureus CGMCC 1.0089, *Listeria monocytogenes* CICC 21635, *Enterococcus faecalis* CICC 10396, *Enterococcus faecium* CGMCC 1.101, *Escherichia coli* CMCC44102) and four indicator fungi (*Aspergillus flavus* CICC 40375, *Rhizoctonia solani* CICC 40529, *Fusarium oxysporum* CICC 2532, *Candida albicans* GGMCC 2.2086) were selected for this experiment. Bacterial inhibition was assessed following the methodology outlined by Liu et al. (2022). The indicator fungi were cultured on potato dextrose agar at 25°C for 5–7 days (Jamyuang et al. 2019). Spore suspensions were collected by adding 15 mL of sterile ultrapure water and quantified using a hematocytometer. The antifungal activity of *Weissella* strains was assessed through an overlay assay as described by Quattrini et al. (2020).

#### 2.3.4 EPS‐Producing Characteristics

Screening of EPS‐producing bacteria was assessed following the methodology outlined by Quattrini et al. (2020). Briefly, bacterial strains were streaked onto MRS‐sucrose agar (60 g/L sucrose) plates and incubated for 48 h at 30°C. EPS production was determined by observing mucoid growth. The EPS yield of *weissella* strains was determined by the alcohol precipitation method, as per the methodology outlined by Yu et al. (2018).

#### 2.3.5 Radical Scavenging Activity

The DPPH and ABTS+ radical scavenging assays were conducted in accordance with the method outlined in a previous publication by Liu et al. (2022), with minor adjustments. Specifically, 100 μL 1 × 10^8^ CFU/mL intact cells (IC) or 100 μL cell‐free supernatant (CFS) were thoroughly combined with 100 μL of a DPPH solution in methanol (200 μM) and incubated at room temperature in the absence of light for a duration of 30 min. Trolox at a concentration of 200 μg/mL was utilized as a positive control. The absorbance was quantified at 517 nm, and the DPPH scavenging capacity was subsequently determined.

$$\text{DPPH scavenging effect}\;\left(\%\right)=\left[\frac{1-A_{517}\left(\text{sample}\right)}{A_{517}\left(\text{blank}\right)}\right]\times 100\%$$

In the ABTS+ radical scavenging assay, 10 μL IC or 100 μL CFS were combined with 100 μL of ABTS+ working solution, subjected to vortexing for 30 s, and subsequently incubated at ambient temperature for 6 min. Trolox, at a concentration of 200 μg/mL, was employed as a positive control. The absorbance was quantified at 734 nm, and the scavenging efficacy of ABTS+ was determined accordingly.

$${\text{ABTS}}^{+}\;\text{scavenging effect}\;\left(\%\right)=\left[\frac{1-A_{734}\left(\text{sample}\right)}{A_{734}\left(\text{blank}\right)}\right]\times 100\%$$

#### 2.3.6 Cholesterol Removal Ability

The cholesterol removal ability was assessed using the o‐phthalaldehyde method as previously described with minor modifications (Feng et al. 1973). MRS‐CHOL broth was prepared by adding cholesterol (Solarbio, Beijing, China) to MRS broth at a concentration of 0.1 g/L. Following inoculation of overnight cultures (1%, v/v) into the MRS‐CHOL broth, incubation was carried out for 24 h at 37°C. Subsequently, the fermentation broth was subjected to centrifugation at 12,857 g for 15 min to eliminate bacteria. Cholesterol content was quantified (Rudel and Morris 1973; Nami et al. 2019), and the percentage of cholesterol removal was calculated accordingly.

$$\text{Cholesterol removal percentage}\;\left(\%\right)=\left(\frac{C_{0}-C_{t}}{C_{0}}\right)\times 100\%$$

*C*_0_: the concentration of cholesterol at the initial medium; *C*_*t*_: the concentration of cholesterol at the end of the inoculation.

#### 2.3.7 Safety Assessment of *Weissella* Strains

##### 2.3.7.1 Antibiotic Susceptibility

In order to evaluate antibiotic resistance, bacterial strains were inoculated at a concentration of 1% (v/v) into MRS broth supplemented with a variety of antibiotics (vancomycin, chloramphenicol, penicillin, streptomycin, gentamycin, kanamycin, erythromycin, ampicillin, and tetracycline) at different concentrations ranging from 2 to 1024 μg/mL. The cultures were then analyzed in triplicate for growth using a microplate reader to measure optical density at 600 nm after a 24 h incubation period at 30°C. (Argyri et al. 2013; Campana, van Hemert, and Baffone 2017).

##### 2.3.7.2 Hemolytic Activity

The hemolytic activity of *Weissella* strains was assessed on Blood Agar supplemented with 7% (v/v) sheep blood (PB001 land bridge, Beijing). Plates were streaked and incubated at 37°C for 48 h. The hemolytic activities of the isolates were determined by the presence of β‐hemolysis, as evidenced by a clear, colorless or lightened yellow zone surrounding the colonies. (Nami et al. 2019).

##### 2.3.7.3 Biogenic Amine Production

Tyrosine (freebase), histidine monohydrochloride, ornithine monohydrochloride, and lysine monohydrochloride (Macklin, Shanghai, China) were utilized as precursor amino acids in this study. The ability of the *Weissella* strains to generate biogenic amines (BA) was assessed following the methodology outlined by Pino, Russo, et al. (2019). The test media were prepared based on the formulation provided by Bover‐Cid and Holzapfel (1999). Each strain was streaked onto the medium and incubated at 37°C for a duration of 4 days. Subsequently, the plates were inspected for the presence of a purple hue in the vicinity of the colonies. Control plates lacking amino acids were utilized for comparison. The experiments were conducted in triplicate.

#### 2.3.8 Statistical Analysis

All sample measurements were done in triplicate. Means and standard deviations were computed, and statistical analysis was conducted using SPSS version 19.0 (IBM Corp., Chicago, IL, USA). A one‐way ANOVA was used to compare means across different treatments, with a significance level set at *p* < 0.05.

## 3 Results

### 3.1 Isolation and Identification

**FIGURE 1:**
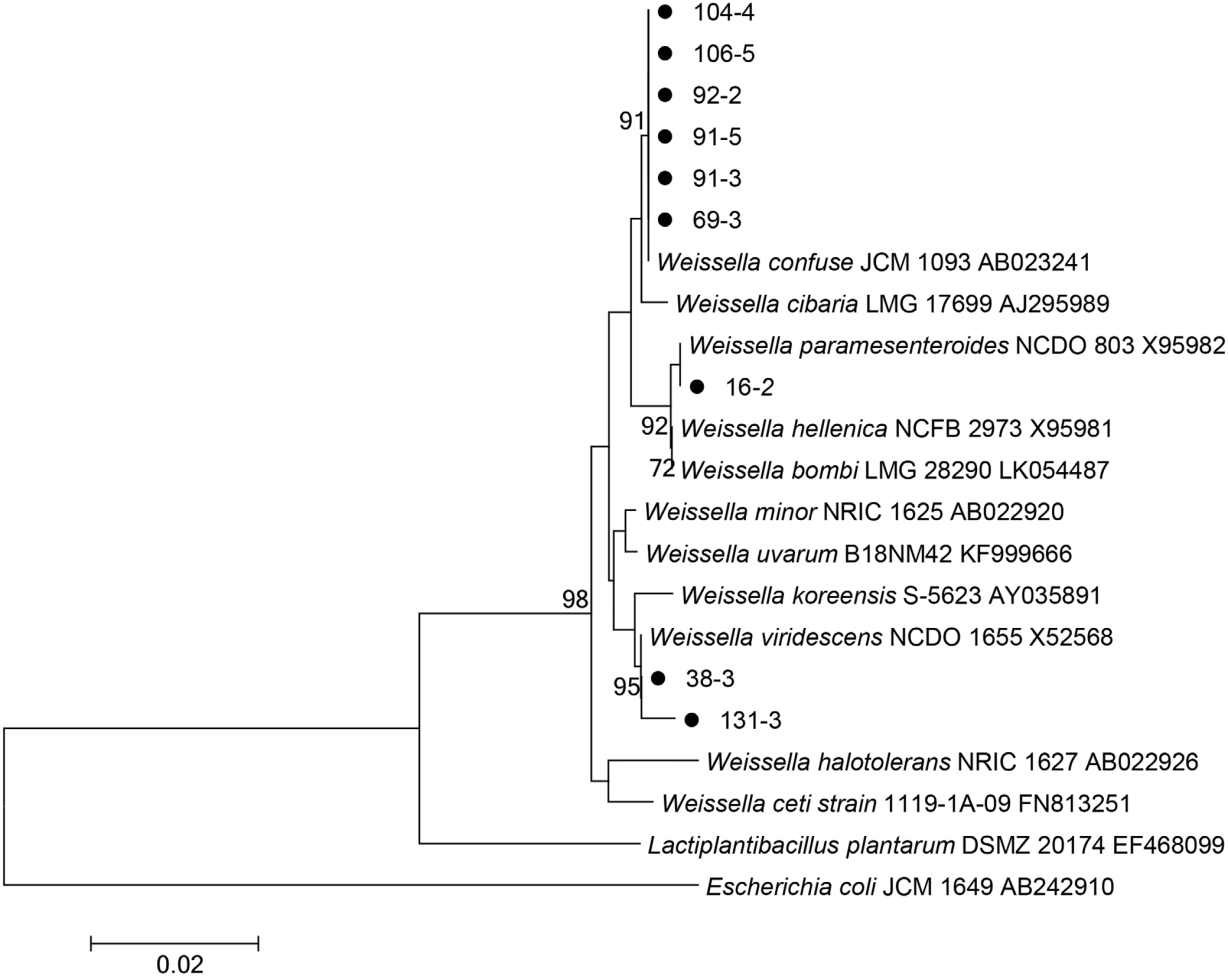
Phylogenetic tree of *Weissella* strains based on neighbor‐joining distance analysis of 16S rRNA gene sequences.

All isolates exhibited characteristics of being Gram‐positive, cocci or rods, facultative aerobes, and gas producers. The PCR products of nine isolates for the 16s rRNA were deemed suitable, with sequencing results detailed in Table 1. A phylogenetic tree illustrating the relationships among the nine *Weissella* strains is presented in Figure 1. Analysis revealed a high degree of similarity, ranging from 98.85% to 100%, between the isolates and known *Weissella* species on NCBI. Specifically, six strains were identified as *W. confusa* , two strains as *W. viridescens* , and one strain as *W. paramesenteroides* .

### 3.2 Acid Production and Growth Properties

**FIGURE 2:**
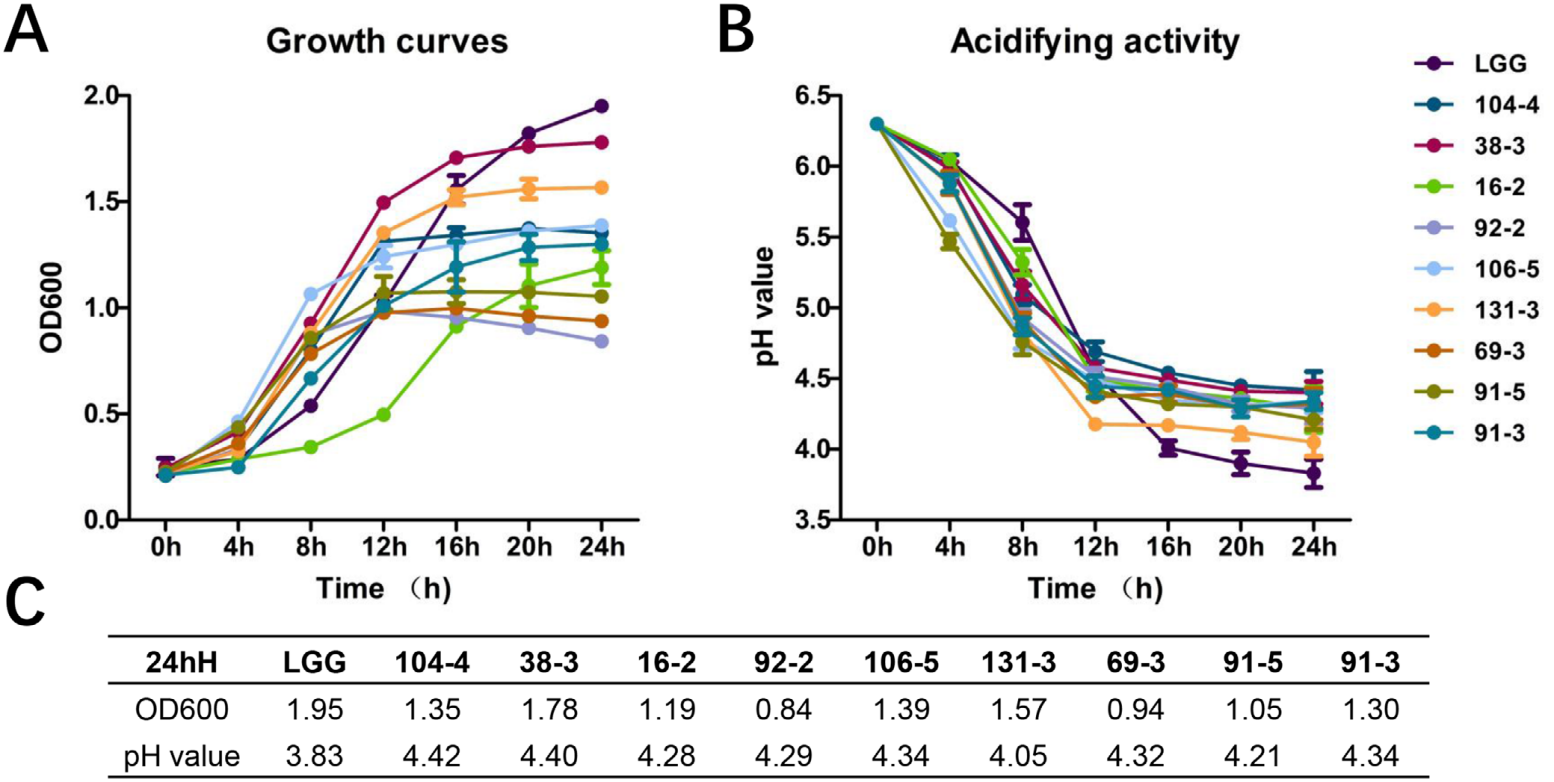
Growth curves (A), acidify activity (B) and OD600/pH value at 24 h (C) of LGG/*Weissella* strains isolated from spontaneous fermented vegetables in Shaanxi, China. Data shown are mean ± SD of triplicate values of independent experiments.

Acid production and growth properties are critical factors to consider when selecting probiotics. The data depicted in Figure 2A indicates that all *Weissella* isolates reached a stable phase after 16 h of incubation, whereas LGG continued to exhibit clear growth trends after 24 h of incubation. Additionally, Figure 2B illustrates that the pH values of all tested strains decreased rapidly during the initial 12 h, followed by a slower decline, which contrasts with the behavior observed in LGG. LGG exhibited the highest acid production capacity and bacterial mass following a 24‐h incubation period, with pH and OD600 values of 1.95 and 3.83, respectively (Table 2). The pH values of *Weissella* fermentation liquor ranged from 4.21 to 4.42, with the exception of strain 131‐3 at 4.05. Moreover, the optical density values at 600 nm of all *Weissella* strains were observed to be lower than that of LGG. These results indicate notable variations in acidification and growth capabilities between *Weissella* strains and LGG, consistent with prior studies (Remize and Fessard 2017). This disparity could potentially be attributed to genus‐specific factors.

**TABLE 2:** Antimicrobial activity of *Weissella* strains from spontaneous fermented vegetables in Shaanxi province, China.

	Bacteria^a^							Fungi^b^			
	*L. monocytogenes* CICC 21635	*E. faecalis* CICC 10396	*E. faecium* CGMCC 1.101	*S. aureus* CGMCC 1.0089	*E. coli* CMCC 44102	*S. flexneri* CMCC 51574	*S. paratyphi* B CMCC 50094	*R. solani* CICC 40529	*C. albicans* GGMCC 2.2086	*Aspergillus flavus* CICC 40375	*Fusarium oxysporum* CICC 2532
104‐4	−	−	−	+	+	+	++	−	−	−	+
38‐3	+	+	−	+	++	++	++	−	−	+	−
16‐2	−	−	−	++	+++	++	++	+	−	−	+
92‐2	−	+	−	+	++	+	−	−	−	−	+
106‐5	−	+	−	+	++	+	+	−	−	+	+
131‐3	++	++	+	++	+++	++	++	+	−	+	+
LGG	++	+	++	++	+++	++	−	−	−	−	+
69‐3	−	−	−	++	++	++	−	−	−	+	+
91‐5	+	+	−	++	++	+	+	+	−	+	+
91‐3	+	−	−	++	++	++	−	−	−	−	+

^a^ Based on inhibition zone (mm), the antibacterial activity of *Weissella* strains was classified as follows: −, no inhibition; +, 0–3; ++, 3–6; +++, > 6.

^b^ The antifungal activity of *Weissella* strains was classified as follows: − no inhibition; + inhibition by using the overlay method. Results of independent experiments (*n* = 3).

### 3.3 Probiotic Properties

#### 3.3.1 Survival Rate in Simulated Gastrointestinal Fluids

**FIGURE 3:**
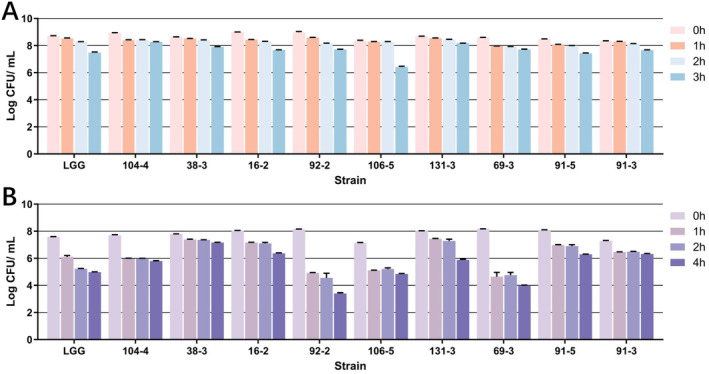
In vitro resistance to simulated gastric fluid (A) and small intestinal fluid (B) of *Weissella* strains isolated from spontaneous fermented vegetables in Shaanxi, China. Data shown are mean ± SD of triplicate values of independent experiments.

After 3 h in simulated gastric fluid, all strains except 106‐5 decreased by about one log CFU/mL. Strain 106‐5 had a larger decrease of about two log CFU/mL. Interestingly, strain 106‐5 remained stable for the first two hours. Survival in simulated small intestine conditions varied among the strains. It was observed that following a 4‐h incubation period in simulated intestinal fluid, strain 38‐3 exhibited the highest survival rate, with strain 91‐3 closely following and experiencing only a minimal reduction (less than 1 log CFU/mL). Strains 16‐2, 91‐5, and 104‐4 demonstrated a reduction range between 1 log CFU/mL and 2 log CFU/mL, while strains 106‐5, 131‐3, and LGG experienced a reduction of 2–3 log CFU/mL. Conversely, strains 92‐2 and 69‐3 displayed a significant decrease in survival rate, with reductions exceeding 4 log CFU/mL, as shown in Figure 3. It is noteworthy that within the simulated intestinal fluid environment, reductions in colony numbers were predominantly observed within the initial hour of incubation.

#### 3.3.2 Cell Surface Characteristics

##### 3.3.2.1 Hydrophobicity

**FIGURE 4:**
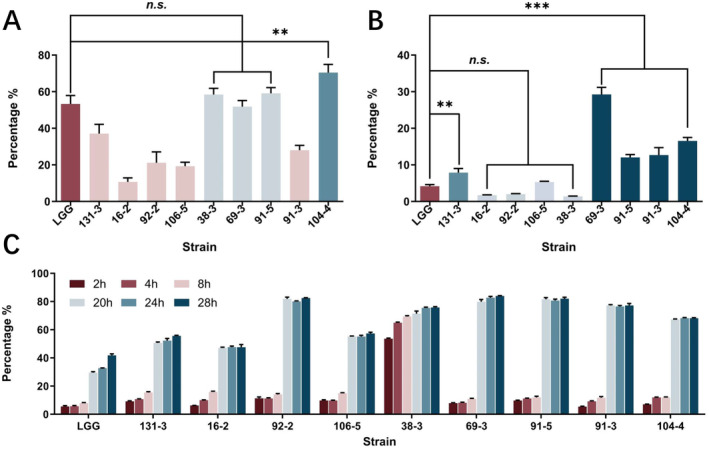
The hydrophobicity (A), adhesion ability to Caco‐2 cells (B), and auto‐aggregation (C) of *Weissella* strains isolated from spontaneous fermented vegetables in Shaanxi province, China. Data shown are mean ± SD of triplicate values of independent experiments, *p* > 0.05, **p < 0.01, ***p < 0.001.

The *Weissella* isolates exhibited varying levels of hydrophobicity, as illustrated in Figure 4A. Following treatment by using chloroform, strain 104‐4 demonstrated the highest hydrophobicity at 70.43%, which was significantly greater than that of LGG at 53.30%. Strains 38‐3, 69‐3, and 91‐5 exhibited similar levels of hydrophobicity (58.41%, 51.85%, 59.07%) to LGG, with no significant differences (*p* > 0.05). In contrast, strains 131‐3, 16‐2, 92‐2, 106‐5, and 91‐3 displayed relatively low cell surface hydrophobicity levels (< 40%) (Zupancic et al. 2019).

##### 3.3.2.2 Adhesion to Caco‐2 Cells

The qualitative assessment of adherence capacity in *Weissella* strains was carried out utilizing the human epithelial Caco‐2 cell line. Comparisons were made between the adhesion ability of *Weissella* strains to Caco‐2 cells and that of the strain LGG (Figure 4B). The results indicated that *Weissella* strains exhibited the ability to adhere to cells following a one‐hour incubation period, with adhesion rates ranging from 1.39% to 29.26%. Specifically, strains 69‐3, 91‐5, 91‐3, 104‐4, and 131‐3 demonstrated significantly higher adhesion values (27.48%, 12.15%, 13.43%, 17.6%, 7.68%) compared to LGG (4.19%). Conversely, the difference between strains 16‐2 (1.69%), 92‐2 (1.98%), 106‐5 (5.53%), 38‐3 (1.38%), and LGG was not significant.

##### 3.3.2.3 Auto‐Aggregation Rate

The auto‐aggregation rate of the *Weissella* strains under evaluation exhibited time‐dependence, as illustrated in Figure 4C. Strain 38‐3 demonstrated high levels of auto‐aggregation at early time points (2 h 53.63%; 4 h 64.92%; 8 h 69.39%), while the auto‐aggregation rates of other strains remained below 20% after eight hours and reached a plateau after 20 h. Specifically, isolates 69‐3 (83.99%), 92‐2 (82.43%), and 91‐5 (82.02%) displayed the highest auto‐aggregation capacities at 28 h. In contrast, LGG exhibited the lowest auto‐aggregation capacity at 28 h, with mean values of 41.77%.

#### 3.3.3 EPS‐Producing Characteristics

**FIGURE 5:**
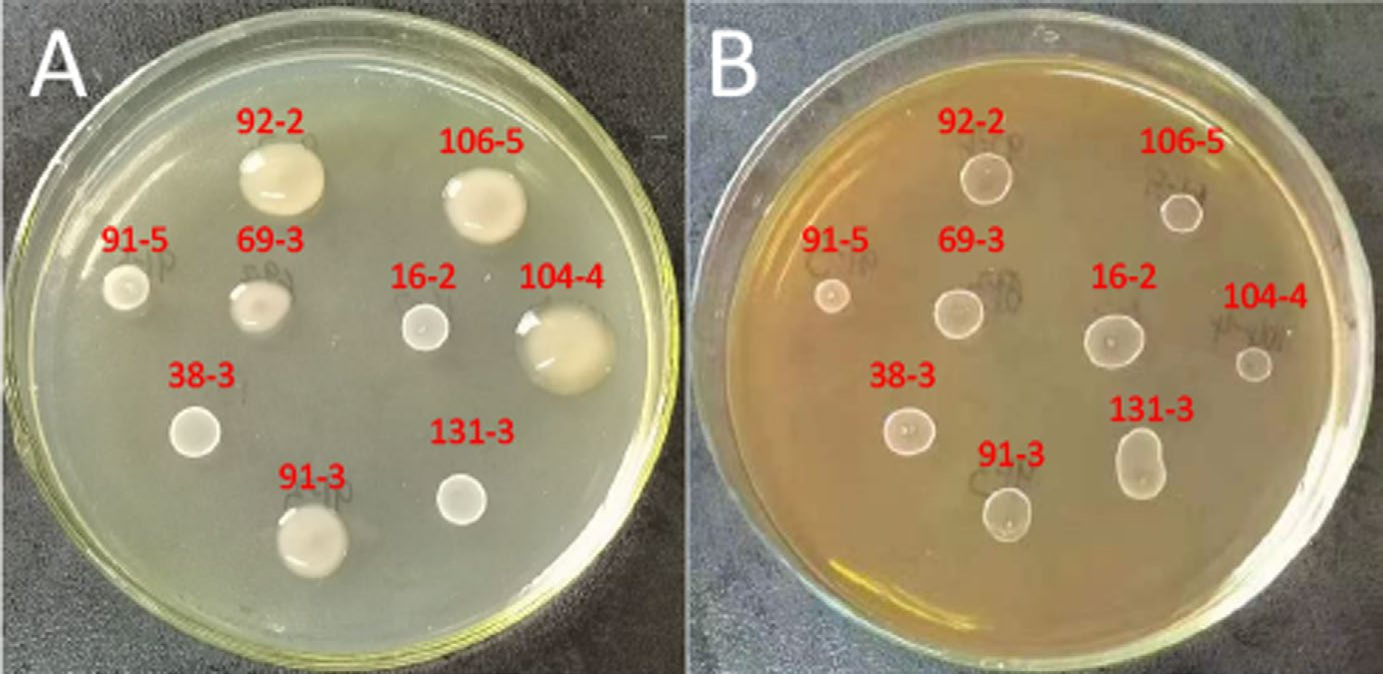
EPS produced by *Weissella* strains isolated from spontaneous fermented vegetables in Shaanxi province, China. EPS production by *Weissella* strains on MRS‐suc (A) and MRS‐glu (B) agar plates after 48 h of incubation at 30°C.

The qualitative screening of EPS production in solid medium MRS‐suc (60 g/L) revealed that six out of nine *Weissella* strains demonstrated the ability to produce EPS, all of which were identified as *W. confuse*. The recorded levels of EPS production for the six *W. confuse* strains, from highest to lowest, were as follows: 104‐4 > 92‐2 > 106‐5 > 91‐3 > 91‐5 > 69‐3 (35.11, 34.99, 9.44, 6.36, 3.39, and 3.22 g/L), as illustrated in Figure 5.

#### 3.3.4 Cholesterol Removal Ability

**FIGURE 6:**
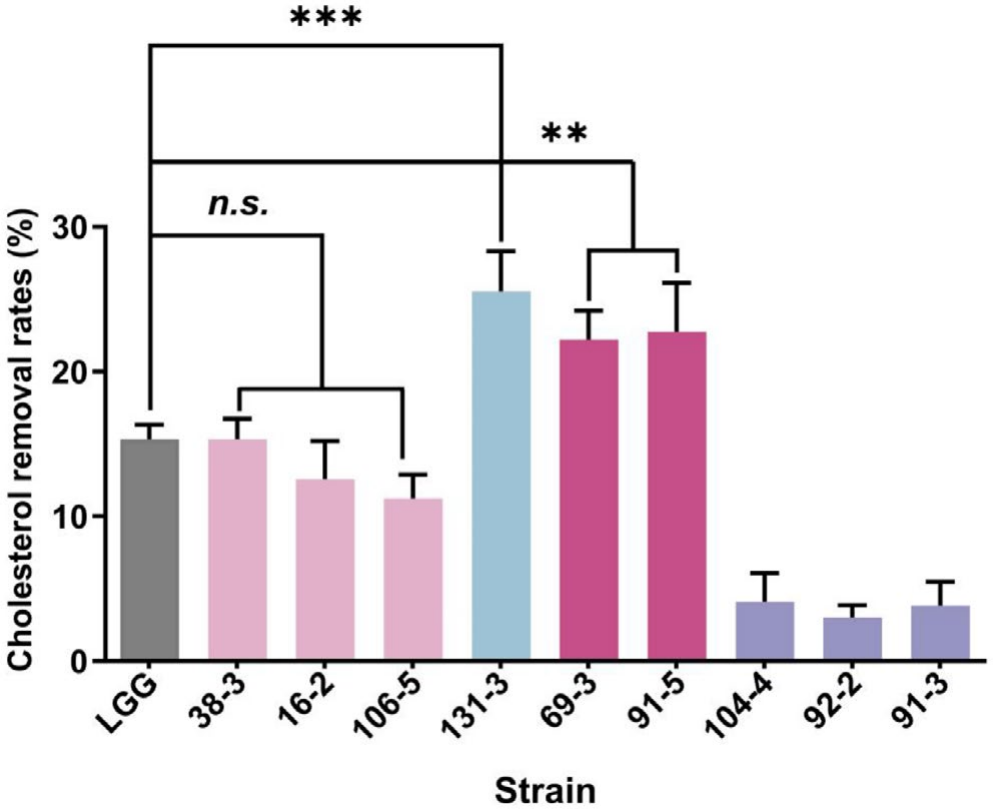
Cholesterol assimilation ability of Weissella strains isolated from spontaneous fermented vegetables in Shaanxi province, China. Data shown are mean ± SD of triplicate values of independent experiments, n.s. *p* > 0.05, **p < 0.01, ***p < 0.001.

Among the nine isolates studied (Figure 6), isolate 131‐3 exhibited the highest cholesterol removal rate at 25.53%, followed by isolates 91‐5 (22.75%), 69‐3 (22.22%), 38‐3 (15.34%), 16‐2 (12.57%), 106‐5 (11.24%), 104‐4 (4.10%), 91‐3 (3.84%), and 92‐2 (3.04%). In comparison to the reference strain LGG, three isolates (38‐3, 16‐2, and 106‐5) demonstrated similar removal rates, while three isolates (91‐5, 69‐3, 38‐3) showed higher removal effects than the reference strain.

#### 3.3.5 Radical Scavenging Activity

**FIGURE 7:**
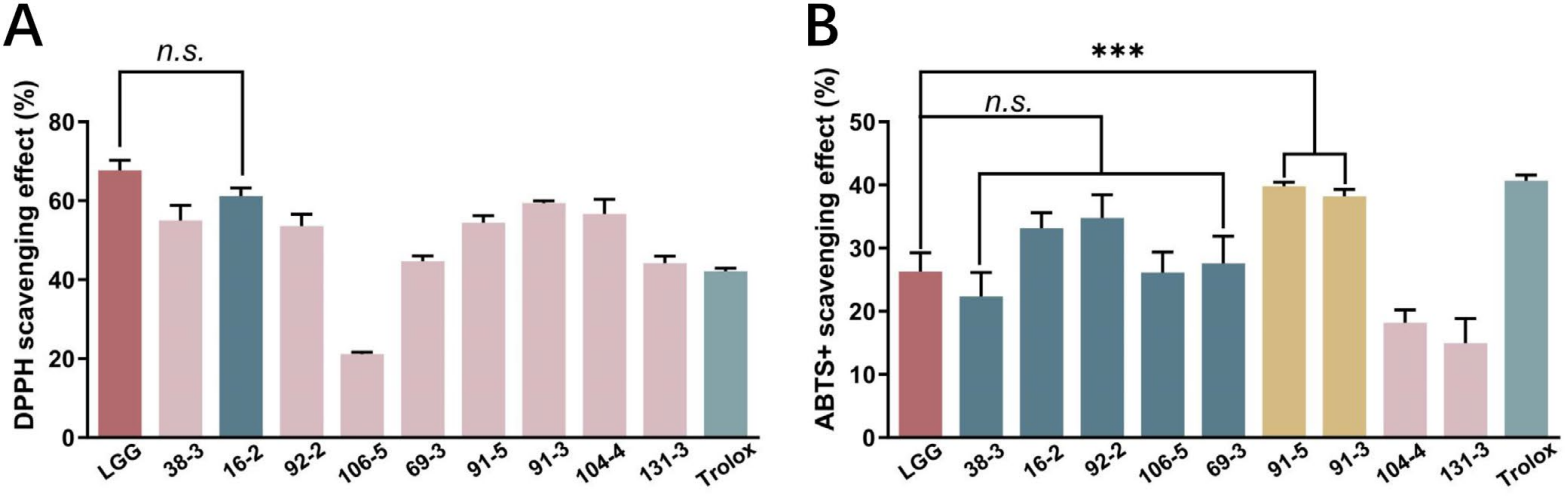
ABTS+ (A) and DPPH (B) scavenging activity of *Weissella* strains isolated from spontaneous fermented vegetables in Shaanxi province, China. Data shown are mean ± SD of triplicate values of independent experiments, n.s. *p* > 0.05, **p < 0.01, ***p < 0.001.

ABTS+ and DPPH reagents are commonly used to assess the free radical scavenging activity of antioxidants. (Sun et al. 2020). The results are shown in Figure 7. Nine strains were tested and exhibited varying degrees of radical scavenging activity. The CFS of the tested strains demonstrated the higher free radical scavenging activity compared to the corresponding IC (Figure S1). In the ABTS+ radical scavenging assay, 38‐3, 16‐2, 92‐2, 106‐5, and 69‐3 exhibited comparable removal levels to LGG (26.31%), while 91‐5, 91‐3 demonstrated notably elevated removal rates (39.82% and 38.20%, respectively) compared to the reference strain. The removal rate of Trolox at a concentration of 200 μg/mL, employed as a positive control, was found to be 40.78%. In the DPPH radical scavenging assay depicted in Figure 7A, it was observed that all samples exhibited DPPH radical scavenging activity, with the majority demonstrating a scavenging rate exceeding 50%. Specifically, sample 16‐2 displayed a scavenging rate of 61.76%, which closely resembled the scavenging rate of LGG at 67.68%, while the DPPH scavenging rate of 200 μg/mL Trolox was measured at 42.15%.

#### 3.3.6 Antimicrobial Activity

**FIGURE 8:**
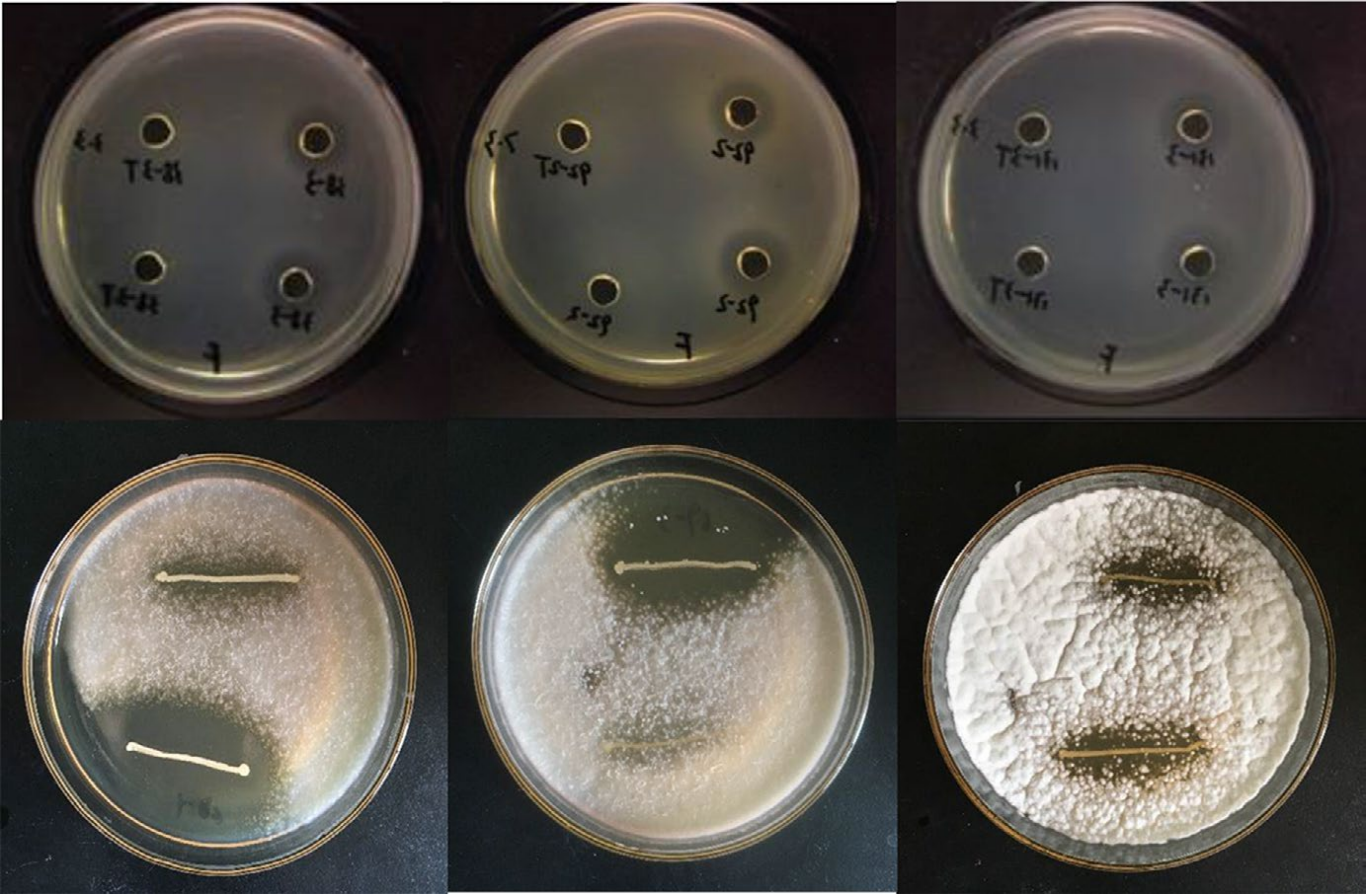
Example of *Weissella* strains antimicrobial activity.

The study evaluated the antagonistic properties of cell‐free supernatants (CFS) derived from *Weissella* strains against a selection of seven foodborne pathogenic bacteria (Figure 8), encompassing both Gram‐negative bacteria and Gram‐positive bacteria. All isolates exhibited inhibitory effects against *S. aureus* , *E. coli* , and *S. fumigatus*. Isolate 131‐1 demonstrated inhibition against all tested pathogens, while isolates LGG and 91‐5 inhibited all six pathogens, with the three lowest pH values of the supernatant recorded at 4.05, 3.83, and 4.21, respectively. The antimicrobial activity of the isolates was observed to be more effective against Gram‐positive bacteria in comparison to Gram‐negative bacteria. Upon adjusting the pH to 5.5, it was observed that none of the strains exhibited inhibition of the pathogenic bacteria, indicating a potential correlation between the inhibitory effect of these strains and the production of organic acids during their growth. Furthermore, four strains of pathogenic fungi were also used as indicators; the isolates demonstrated no inhibitory effects on *F. oxysporum*, a fungus known to thrive in acidic conditions, while displaying diverse antagonistic activities against *R. solani* and *A. flavus* , as outlined in Table 2.

### 3.4 Safety Evaluation

**FIGURE 9:**
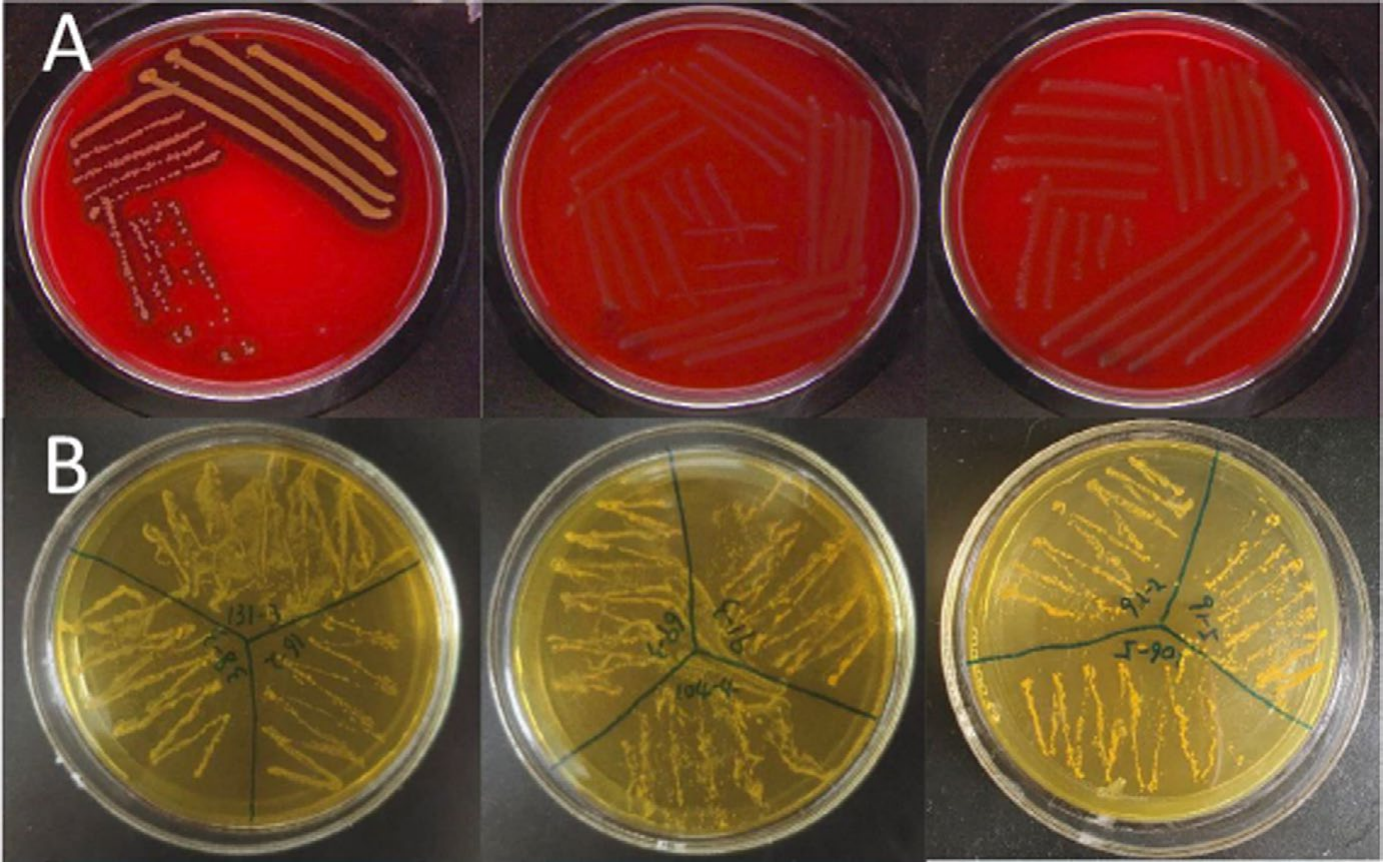
Hemolytic activity of *Staphylococcus aureus* CGMCC 1.0089 and *Weissella* strains (A); Biogenic amine production of *Weissella* strains (B) isolated from spontaneous fermented vegetables in Shaanxi province, China.

To provide additional information about the safety profile of the test strains, assessments of their hemolytic activity and BA production were conducted. The results, depicted in Figure 9, indicated that all *weissella* isolates exhibited no hemolytic activity and BA production.

In order to provide additional information on the safety of the test strains, their hemolytic activity and biogenic amine production were evaluated. The results are shown in Figure 9, indicating that all isolates were free of hemolytic activity and biogenic amine production.

In addition to these safety characteristics, the antibiotic susceptibility of the strains was also investigated. Table 3 presents the minimum inhibitory concentrations of *Weissella* strains and LGG toward antibiotics belonging to distinct categories. Strains were defined as resistant when their inhibition concentration exceeded the established cut‐off value (Aquilina et al. 2012). The findings indicate that all isolates exhibited resistance to vancomycin and susceptibility to ampicillin, erythromycin, tetracycline, chloramphenicol, and clindamycin, aligning with prior research (Lee et al. 2012; Liu et al. 2022) suggesting that *Weissella* spp. possess inherent resistance to vancomycin, similar to other lactic acid bacteria. (Bhagat et al. 2020; Pino, Bartolo, et al. 2019). The findings also indicate that kanamycin, streptomycin, and gentamicin show weak inhibitory effects on *Weissella* strains. All test strains have kanamycin resistance except for 38‐3, 16‐2, and 106‐5. The inhibitory concentration of streptomycin reached up to 256 mg/L. Additionally, isolates 92‐2 and 131‐3 exhibited resistance to gentamicin, while the remaining isolates did not. In conclusion, isolates 92‐2 and 131‐3 showed resistance to two antibiotics, while isolates 104‐4, 69‐3, 91‐5, 91‐3, and the reference strain LGG were resistant to one antibiotic. Isolates 38‐3, 16‐2, and 106‐5 displayed non‐susceptibility to all tested antibiotics.

**TABLE 3:** Antibiotic susceptibility of Weissella strains from spontaneous fermented vegetables in Shaanxi province, China.

	A	E	K	S	T	V	Ch	G	Cl	Ci	Ce
104‐4	< 2	< 2	256^R^	64	< 2	512	2	8	< 2	2	2
038‐3	2	< 2	32	64	< 2	512	2	8	< 2	< 2	16
016‐2	< 2	< 2	64	16	< 2	256	2	2	< 2	2	< 2
092‐2	< 2	< 2	≥ 1024^R^	256	< 2	512	2	32^R^	< 2	< 2	2
106‐5	< 2	< 2	32	16	< 2	512	2	4	< 2	< 2	< 2
131‐3	2	< 2	512^R^	256	8	512	< 2	64^R^	< 2	128	2
LGG	4	< 2	256^R^	16	< 2	≥ 1024	2	4	< 2	256	16
069‐3	2	< 2	512^R^	256	< 2	512	2	8	< 2	< 2	< 2
091‐5	< 2	< 2	128^R^	< 2	< 2	512	2	16	< 2	< 2	< 2
091‐3	< 2	< 2	128^R^	32	< 2	512	2	16	< 2	2	< 2

*Note: Weissella* strains resistant according to the EFSA's breakpoints (EFSA 2012).

Abbreviations: A, ampicillin; Ce, cefixim; Ch, chloramphenicol; Ci, ciprofloxacin; Cl, clindamycin; E, erythromycin; G, gentamicin; K, kanamycin; S, streptomycin; T, tetracycline; V, vancomycin.

## 4 Discussion

This study evaluates the probiotic potential of nine *Weissella* strains through a series of in vitro experiments, including survival in the gastrointestinal tract, cell surface characteristics, free radical scavenging capacity, antimicrobial activity, production of exopolysaccharides, and assimilation of cholesterol. Additionally, safety assessments were conducted on all *Weissella* strains, focusing on hemolytic activity, production of biogenic amines (BAs), and susceptibility to antibiotics, due to concerns regarding the safety of *Weissella* spp.

The fulfillment of the efficacy of probiotics hinges on their capacity to endure, traverse, and establish residence in the gastrointestinal tract, necessitating a primary assessment of their tolerance to the harsh conditions of the GI tract, including low pH levels and the presence of bile salts. Previous studies have indicated that *Weissella* strains exhibit robust survival rates under low pH conditions (Anandharaj and Parveen Rani 2014; Lee et al. 2012). In comparison to the reference strain LGG, most *Weissella* strains displayed higher survival rates after 3 h of incubation in simulated gastric fluid, with the exception of strain 106‐5. Consistent with prior studies, *Weissella* strains originating from fermented plant‐based foods exhibit notable efficacy in resisting acidic conditions. The survival rates of *Weissella* strains in simulated intestinal fluid demonstrate significant variability. Among the nine strains tested, seven exhibited higher survival rates than LGG, indicating the favorable survival potential of *Weissella* strains in the intestinal environment.

Probiotic strains with promising potential should demonstrate the ability to adhere to intestinal epithelial cells and resist elimination from the large intestine by peristalsis. This capability is influenced by a range of surface properties, such as cell adhesion, cell surface hydrophobicity, and auto‐aggregation ability. Attachment to the intestinal mucosa is crucial for enhancing defenses against infection by intestinal pathogens. Our findings indicate that strains 69‐3, 91‐5, 91‐3, 104‐4, and 131‐3 exhibited significantly greater adhesion compared to LGG.

Hydrophobicity is a critical characteristic of cell surfaces that is closely linked to the adhesion capacity of probiotic bacteria to epithelial cells (Ayyash et al. 2018). Specifically, strains 104‐4, 38‐3, 69‐3, and 91‐5 exhibit high levels of cell surface hydrophobicity (> 40%), which may not directly correspond to their adhesion capabilities. This discrepancy could be attributed to the influence of various factors such as temperature, pH value, and the composition of the culture medium on the properties of cell adhesion. Additionally, previous research has also failed to establish a clear correlation between hydrophobicity and adhesion in probiotic bacteria (García‐Cayuela et al. 2014).

Auto‐aggregation is a significant property of probiotics that facilitates bacterial colonization, recognition, communication, and survival, enabling them to achieve a high cell density in the gastrointestinal tract and potentially serve as a barrier against pathogenic bacteria. In comparison to LGG, all tested strains exhibited greater auto‐aggregation values at each time interval.

EPS plays a crucial role in facilitating colonization by promoting bacterial adhesion and aggregation, enhancing tolerance to environmental stress, and providing benefits to humans (Sorensen et al. 2022). Consequently, the production of EPS serves as a significant indicator for probiotics. The ability to produce EPS is a distinguishing phenotypic characteristic of the *Weissella* genus (Kavitake, Devi, and Shetty 2020). Our study revealed that all strains of *Weissella* confuse exhibit EPS production capabilities, with strain 104‐4 demonstrating an impressive EPS yield of 35.11 g/L, surpassing values reported in most existing literature (Feng et al. 2018; Rosca et al. 2018; Yu et al. 2018). In recent years, *W*. confuse has garnered considerable interest for its capacity to generate substantial quantities of dextran, fructan, and heteropolysaccharides, as well as unique non‐digestible oligosaccharides with prebiotic properties that can mitigate infection and diarrhea, improve gut metabolic functions, and selectively promote beneficial intestinal bacteria. Consequently, additional research is warranted to investigate the compositions and functionalities of the exopolysaccharides produced by these isolates.

The antimicrobial activity of probiotics is a crucial characteristic that serves as a natural defense mechanism against harmful bacteria (Bajic et al. 2019). This research utilized foodborne pathogenic bacteria and fungi as indicators to assess the antibacterial properties of *weissella* strains. The findings suggest that organic acids may play a significant role in the antibacterial activity of these strains, and acidic conditions may enhance the efficacy of their bacteriostatic substances (Rocha‐Ramírez et al. 2021). Notably, the supernatants of strains 131‐1, LGG, and 91‐5 exhibited the lowest pH values, with strain 131‐1 demonstrating inhibition of almost all tested pathogens and strain 91‐5 inhibiting nine different pathogens. However, the inhibitory effect on the growth of pathogenic bacteria was significantly diminished when the pH value was adjusted to 5.5. The findings indicate that the inhibitory effect is not only dependent on the quantity of organic acid produced but also on the specific species of organic acid (Somashekaraiah et al. 2019). In comparison to LGG, *Weissella* strains that ferment glucose produce a wider range of organic acids, such as 38‐3, which have a pH value of 4.4 in the supernatants and exhibit inhibitory activity against six indicator bacteria. Although 106‐5 and 91‐3 have similar pH values at 24 h, there are distinct differences in their antibacterial spectrum. However, further data is necessary to substantiate the validity of this initial conclusion.

Reactive oxygen species (ROS) can arise from endogenous oxidative metabolism or exposure to exogenous pro‐oxidants (Abriouel et al. 2015). Excessive production of ROS can lead to oxidative stress, a condition associated with neurodegenerative diseases (Miguel‐Gordo et al. 2019). Previous research (Liu et al. 2022) has demonstrated that many strains of lactic acid bacteria have the ability to mitigate oxidative stress by scavenging free radicals. In this study, we utilized two methods, DPPH and ABTS+ radical scavenging activity, to assess the antioxidant capacity of *Weissella* CFS, aiming to overcome the constraints of relying on a single antioxidant property test (Cao et al. 2019; Ragul et al. 2020). This study demonstrated significant variation in the DPPH and ABTS+ scavenging rates among different strains of *Weissella*, showing strain‐specific differences. The scavenging effect (%) of DPPH radicals was found to be higher than that of ABTS+ radicals, suggesting a stronger scavenging capacity of the strains for DPPH radicals. This finding is consistent with previous research (Zhang et al. 2020). Furthermore, the radical scavenging capacity of *Weissella* IC was evaluated, revealing lower DPPH and ABTS+ scavenging rates compared to CFS, consistent with the literature (Cao et al. 2019).

Elevated cholesterol levels have been associated with various health risks, including cardiovascular disease, metabolic syndrome, and type 2 diabetes (Chen et al. 2016). Prior research has demonstrated the cholesterol‐lowering properties of fermented foods containing Lactobacillus spp. or Bifidobacterium spp. (Khalil et al. 2018), but investigations into the cholesterol‐lowering effects of *Weissella* spp. are scarce. This study aims to assess the cholesterol‐reducing capabilities of nine *Weissella* strains in vitro. The findings suggest that the capacity for cholesterol removal varies among different strains. Five isolates exhibited similar (*p* > 0.05) or greater (*p* < 0.05) cholesterol removal capabilities compared to the reference strain. Previous studies have established a notable correlation between the in vitro and in vivo cholesterol removal abilities (Wang et al. 2012). Strains 91‐5, 69‐3, and 38‐3, which demonstrated high cholesterol assimilation in vitro, show promise as potential agents for lowering cholesterol levels (Shen et al. 2016).

The absence of hemolytic activity and antibiotic resistance is regarded as a fundamental safety requirement for the identification of a probiotic strain (FAO/WHO 2002). A few studies have identified lactic acid bacteria (LAB) as the primary producers of biogenic amines (BAs) in fermented foods (Barbieri et al. 2019). None of the tested strains exhibited β‐hemolytic activity or produced biogenic amines. Additionally, in accordance with EFSA guidelines (EFSA 2012), the susceptibility of the *Weissella* strains to various antibiotic groups was assessed.

In recent years, there has been a notable rise in bacterial resistance to commonly used antibiotics, primarily attributed to the overuse and misuse of these medications. This phenomenon poses considerable risks and challenges for patients suffering from infections. Various studies have indicated that not only pathogenic strains but also symbiotic bacteria, such as lactic acid bacteria (LAB), may serve as significant reservoirs for antibiotic resistance genes. This raises concerns about the potential transfer of resistance genes from LAB to human pathogens in the intestinal microbiota, leading to potentially severe outcomes.

In the last decade, extensive research has been conducted on antibiotic resistance in lactic acid bacteria, leading to the reevaluation of the Generally Recognized as Safe (GRAS) status of *Enterococcus*. Consequently, the criterion of “safe use history” alone is insufficient for ensuring safety, necessitating the evaluation of probiotics according to global safety standards. In the absence of defined Minimum Inhibitory Concentration (MIC) breakpoints for the *Weissella* genus by the European Food Safety Authority (EFSA), the antibiotic resistance of *Weissella* spp. strains was compared to that of the commercially available strain LGG. The MIC breakpoints of *Leuconostoc*, a closely related phylogenetic relative, were employed as the reference standard in this investigation.

There are differing perspectives on the mechanisms behind the resistance of *Weissella* spp. to aminoglycoside antibiotics, with one prevalent theory proposing that enzymatic modification of the antibiotic is the key factor in bacterial aminoglycoside resistance. Aminoglycoside‐modifying enzymes (AME) facilitate the covalent modification of amino or hydroxyl functional groups, resulting in reduced binding efficiency to ribosomal RNA and enabling bacterial survival. Despite all isolates displaying relatively high levels of aminoglycoside resistance, there was notable variability in the survival rates among different strains. The inhibitory concentrations of kanamycin, streptomycin, and gentamicin ranged from 32 to 1024, 2 to 256, and 2 to 24, respectively. The variability in inhibitory concentrations may be attributed to variations in the levels of AME enzymes produced by different strains. It is important to note a limitation in this research, as antimicrobial resistance profiles were compared with a commercial probiotic LGG, as cut‐off values for *Weissella* strain are currently unavailable.

The conventional perspective posits that probiotic strains deemed promising should not exhibit antibiotic resistance. However, instances of antibiotic resistance have been documented in certain Generally Recognized as Safe (GRAS) lactic acid bacteria strains utilized in starter cultures, dairy products, and probiotic supplements (Zheng et al. 2017). The commercial probiotic LGG employed in this study also demonstrates kanamycin resistance. An alternative viewpoint proposes that the antibiotic resistance profiles of probiotics are crucial for their viability in the gastrointestinal tract during antibiotic therapy, which in turn have a stabilizing effect on the intestinal metabolic homeostasis (Kang et al. 2019). However, the premise is that antibiotic resistance must be “specific” and “intrinsic.” Therefore, a strain‐specific safety evaluation is necessary when selecting *Weissella* spp. as a probiotic.

The current practical reference for food culture in practice is the “IDF List 2002,” published jointly by the International Dairy Federation (IDF) and the European Food and Feed Culture Association (EFFCA) in 2002 (Kang et al. 2019). This list is based on historical usage of microorganisms in food before 1958, deemed safe due to their long history of use. However, as the focus was primarily on commercially available dairy cultures, there was an unmet need for a list with a broader scope. Bourdichon et al. (2012) have introduced an updated compilation titled “microorganisms with technologically beneficial use in food fermentations,” which encompasses various food matrices such as dairy, meat, fish, vegetables, legumes, cereals, beverages, and vinegar. This inventory includes nine species of *Weissella* identified as valuable microorganisms for food fermentation processes.

*Weissella* spp. is involved in the fermentation process with *Lactobacillus, Leuconostoc, Lactococcus*, and *Pediococcus* (Kang, Yeu, and Hong 2019). Over the past decade, many studies have been conducted on the probiotic properties of *Weissella*, and this class of strains is known to have a variety of beneficial effects, such as antioxidant, cholesterol‐lowering, immunomodulatory, and anticancer (Fairfax, Lephart, and Salimnia 2014; Jang et al. 2016; Li et al. 2017; Pabari et al. 2020; Teixeira et al. 2021; Wang et al. 2017). *W. cibaria* CMU and CMS1 have been registered as safe raw materials by the Korea Food and Drug Administration (KFDA) and actively promoted the commercialization of these strains as oral care probiotics in Korea (Kang, Yeu, and Hong 2019). However, the *Weissella* strain didn't realize large‐scale application, as it is not (yet) FDA‐GRAS certified. In order to establish its GRAS status, further evaluation of its safety and functionality should be conducted according to international standards.

## 5 Conclusions

This research represents the first investigation into the safety and functional characteristics of *Weissella* strains derived from naturally fermented vegetables in Shaanxi province. The safety evaluation revealed that the antibiotic susceptibility profiles of strains 69‐3, 91‐3, 91‐5, 104‐4, 106‐5, 16‐2, and 38‐3 were comparable to or exceeded that of the LGG. Additionally, hemolytic and ammonia production properties were assessed, with no positive outcomes observed. When considering the probiotic attributes, strain 91‐5 exhibited the most promising potential as a probiotic microorganism, while strain 104‐4 demonstrated the highest yield of EPS. All these results demonstrate that 91‐5 and 104‐4 could be further used as probiotic candidates for new functional food supplements. Further research on *Weissella* spp. is warranted. Shaanxi spontaneous fermented vegetables offer a potential source of novel probiotics. Our study has verified that the nine strains of *Weissella* are compliant with international safety standards and have the potential to attain Generally Recognized as Safe (GRAS) status. It is anticipated that upon receiving GRAS approval, these strains will be utilized globally as a key ingredient in a variety of functional and fermented food products.

## Author Contributions

**Chen Liu:** conceptualization (lead), formal analysis (lead), validation (equal), writing – original draft (lead). **Chao An:** data curation (equal), methodology (equal), writing – review and editing (supporting). **Jingjing Zhang:** validation (equal), writing – review and editing (equal). **Yao Liu:** data curation (supporting), validation (supporting), writing – review and editing (supporting). **Qiwen Zhang:** validation (supporting). **Hao Ding:** data curation (supporting), methodology (supporting). **Saijian Ma:** methodology (supporting). **Wenjiao Xue:** conceptualization, writing – review and editing (supporting).

## Conflicts of Interest

The authors declare no conflicts of interest.

## Supporting information


Figure S1.

## Data Availability

Data are available on request.
