# Supplementary material for: Evaluation of Safety and Probiotic Properties of Weissella spp. in Fermented Vegetables From Xi'an, Shaanxi, China
Source: Food Sci Nutr. 2024 Dec 2;13(1):e4592. doi: 10.1002/fsn3.4592 (PMC11717038; doi:10.1002/fsn3.4592)
Supplement: Supplementary file 1 — Figure S1. [file FSN3-13-e4592-s001.docx]

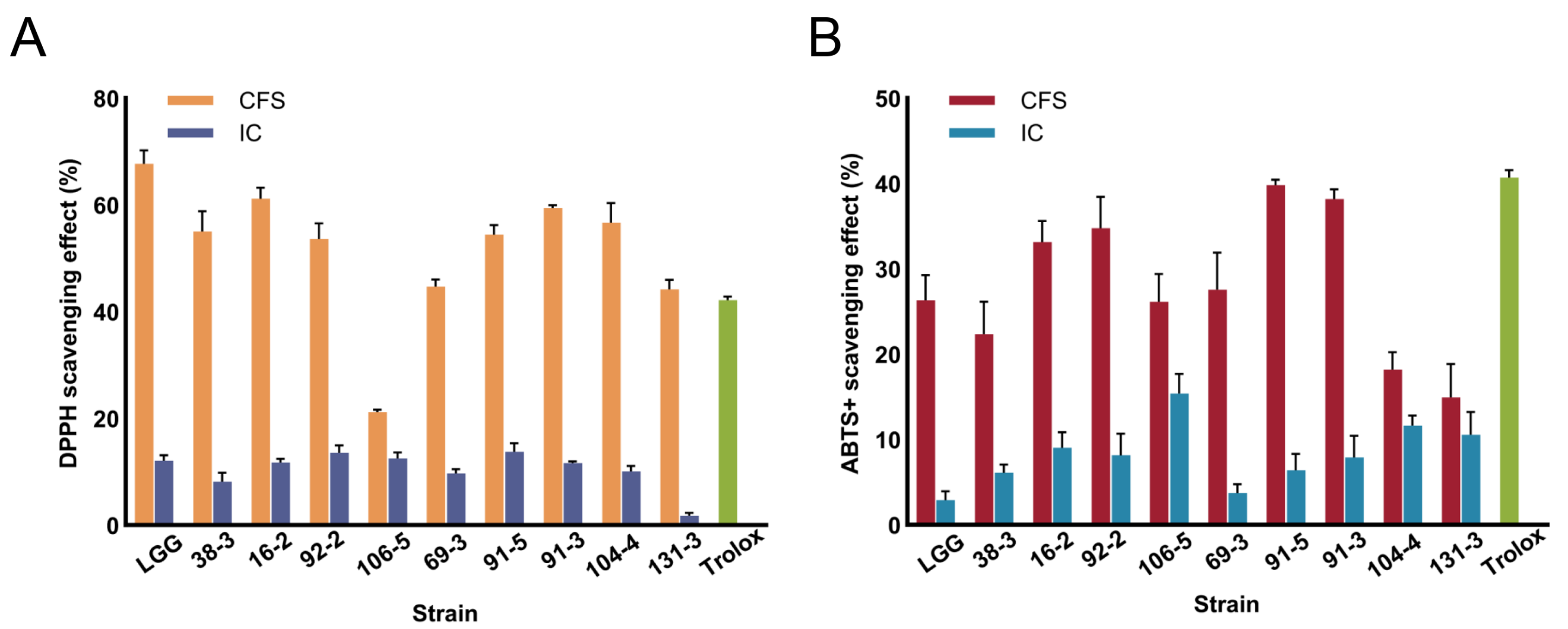


**Figure S1.** DPPH (A) and ABTS+ (B)scavenging activity of *Weissella* strains isolated from spontaneous fermented vegetables in Shaanxi province, China. CFS: cell-free supernatant；IC: intact cells.
